# Comparative efficacy of portable active cooling vs. passive cooling for reducing core temperature in exertional heat stroke: a systematic review and meta-analysis

**DOI:** 10.3389/fpubh.2026.1811173

**Published:** 2026-05-29

**Authors:** Chuankai Shi, Taimin Zhang, Li Xia, Wenhui Shi, Jinquan Qu, Laiyang Song, Jiangwei Liu

**Affiliations:** 1 Department of Graduate School, Xinjiang Medical University, Urumqi, China; 2 Desert Medicine Laboratory, General Hospital of Xinjiang Military Command, Urumqi, China; 3 Xinjiang Medical University, Urumqi, China; 4 Department of External Medicine II, Xinjiang Corps Hospital of the Chinese People's Armed Police Force, Urumqi, China

**Keywords:** cooling rate, cooling vest, exertional heat stroke, meta-analysis, portable cooling

## Abstract

**Background:**

Exertional heat stroke (EHS) and exercise-induced hyperthermia require rapid reduction of core temperature (Tc). Portable active cooling devices are uesed in field settings where cold-water immersion (the gold standard) is not feasible, but their efficacy compared to passive cooling remains unclear.

**Objective:**

This review and analysis compared cooling rates between portable active cooling and passive cooling.

**Methods:**

We performed a systematic review and meta-analysis (PRISMA 2020, PROSPERO: CRD420261292698) of randomized and crossover trials. Eligible studies compared portable active cooling (type A: external refrigerant-based cooling systems; type B: wearable pre-cooling systems) with passive cooling in adults with exercise-induced hyperthermia. The primary outcome was mean Tc cooling rate (°C/min), analyzed using a random-effects model.

**Results:**

11 trials (*n* = 148 participants) were included. Portable active cooling produced a significantly faster cooling rate than passive cooling (MD = 0.02 °C/min, 95% CI: 0.01 to 0.03, *p* < 0.00001). Subgroup analysis revealed that type A devices had superior cooling efficacy (MD = 0.04 °C/min, 95% CI: 0.02–0.04), particularly when ambient temperature exceeded 30 °C. In contrast, type B devices showed no significant advantage (MD = 0.00 °C/min).

**Conclusions:**

For field management of exertional heat stroke or severe exercise-induced hyperthermia, portable active cooling systems that use external refrigerant-based cooling (type A) are more effective than passive cooling. Wearable pre-cooling systems (type B) do not demonstrate superior efficacy. Selection of cooling devices should prioritize cooling mechanism over mere portability.

**Systematic review registration:**

https://www.crd.york.ac.uk/PROSPERO/view/CRD420261292698, identifier: CRD420261292698.

## 1 Introduction

Exertional heat stroke (EHS) is a life-threatening emergency caused by intense physical exertion. It is defined clinically by a core body temperature exceeding 40 °C accompanied by central nervous system dysfunction, including manifestations such as disorientation, seizures, or coma (1, 2). In the context of global climate change and the escalating prevalence of high-intensity physical activities, the incidence of EHS has demonstrates a concerning upward trajectory, emerging as a substantial public health concern particularly among athletes, military personnel, and outdoor laborers (3, 4).

The pathophysiological of EHS involves more than thermoregulatory failure; it includes a cascade of molecular and systemic events. EHS is fundamentally an acute systemic inflammatory response and multiple organ dysfunction syndrome (MODS) triggered by thermal cytotoxicity (2, 5). Critically elevated core temperatures (Tc) (typically > 40.5 °C) cause direct cellular injury through protein denaturation, altered membrane fluidity, and mitochondrial dysfunction (6). At the same time, thermoregulatory redistribution of visceral blood flow and resulting intestinal ischemia compromise the mucosal barrier, facilitating endotoxin translocation into the systemic circulation (7, 8). This process lead to a pronounced inflammatory response and coagulopathy, cresting a self-perpetuating cycle that can rapidly progress to disseminated intravascular coagulation (DIC), rhabdomyolysis, and multi-organ failure (9–12). In EHS, mortality rates surpassing 30% have been documented as a consequence of this uncontrolled pathophysiological cascade (13).

Therefore, rapid and effective temperature reduction is the cornerstone of EHS management. This intervention not only addresses hyperthermia itself but, more importantly, interrupts the pathophysiological cascade during the transition from “heat toxicity” to “inflammatory storm.” Current clinical guidelines emphasize initiating cooling within the “golden 30-min window” to significantly reduce mortality and morbidity (14, 15). However, several challenges remain. The “cooling-shivering paradox” may reduce cooling rates and potentially worsen metabolic disturbances, thereby increasing the risk of MODS (16). Additionally, the frequent coexistence of coagulopathy and bleeding tendency requires tailored organ support, and optimal cooling protocols are still debated. Although whole-body cold-water immersion is the gold-standard method because its high cooling efficiency, its use in pre-hospital, field, or resource-limited settings is often limited by logistical barriers, patient access issues, and safety concerns (17, 18).

In this context, portable active cooling devices emerged as promising alternative or adjunctive therapeutic options. These devices fall into two categories. Type A (externally sourced cooling systems) includes ice-water-soaked waterproof sheets or circulating cold-water blankets, which use conductive and/or evaporative mechanisms for large-area cooling. Type B (wearable pre-cooled systems) includes phase-change material vests or chemical cooling garments, which rely on stored internal cooling capacity for localized conductive cooling. Passive cooling measures, such as moving to shaded areas, removing excess clothing, and resting, remain the most basic initial intervention (19).

Previous studies compared various cooling modalities, but results on their relative efficacy are inconsistent, particularly regarding the cooling rate of core body temperature—a key determinant of clinical outcomes. Systematic evidence is limited. Moreover, active cooling devices operating on different principles may have fundamental performance differences, which directly affect their ability to achieve the rapid cooling required by EHS pathophysiology, and thus influence clinical and field-based device selection.

Consequently, this systematic review and meta-analysis synthesizes existing clinical trial data to compare the effectiveness of portable active cooling devices vs. passive cooling in lowering core body temperature in EHS patients, with the primary outcome being the mean core cooling rate. In addition to this therapeutic comparison, this study explores underlying thermoregulatory mechanisms and implementation considerations through subgroup analyses, aiming to provide both evidence-based recommendations and physiological insights for EHS emergency management in pre-hospital and resource-limited settings.

## 2 Methods

The present investigation was rigorously performed in accordance with the 2020 PRISMA guidelines to maximize methodological transparency and ensure reproducibility. The study protocol was prospectively registered in the PROSPERO international prospective register of systematic reviews (Registration ID: CRD420261292698) (20).

### 2.1 Information sources and search strategy

To ensure a comprehensive literature search, two investigators independently performed systematic searches across multiple electronic databases, including PubMed, Web of Science, Cochrane Library, Embase, and MEDLINE. The search period encompassed records from each database's inception to January 20, 2026. Any disagreements between the two investigators were resolved by consultation with a third researcher.

The search strategy employed a combination of MeSH terms and free-text keywords connected by Boolean logical operators (“AND” and “OR”). The search framework was structured around three key domains: (1) disease/condition—exercise-induced heat stroke and related terms (e.g., “Heat Stroke,” “Exertional Heat Stroke”); (2) intervention—portable active cooling devices (including waterproof cloth-assisted cooling, cooling vests, and related synonyms); and (3) study design—randomized controlled trials and crossover trials. The detailed PubMed search strategy is available in Supplementary material 1. No restrictions were applied on language or country of publication. Reference lists of included studies and relevant systematic reviews were also manually searched.

### 2.2 Eligibility criteria

Studies were selected based on the PICOS framework with the following criteria: (1) Participants were adolescents or adults (≥12 years) with elevated core body temperature (typically >38.5 °C) included by exercise or controlled heat exposure, including healthy volunteers, athletes, and military personnel. Most included studies induced moderate hyperthermia (38.0–39.5 °C) in healthy individuals rather than clinical exertional heat stroke (Tc > 40.0 °C with central nervous system dysfuction); this limitation is addressed in the discussion. Studies on classical heatstroke or fever from infection or medication were excluded. (2) Interventions were portable active cooling devices. Type A devices (external refrigerant-based cooling systems) used a continuous external refrigerant supply (e.g., ice, ice-water mixtures, circulating cold water) for conductive and/or evaporative cooling, including ice-water-soaked waterproof/canvas materials, ice sheets, and circulating cold water blankets. Type B devices (wearable pre-cooling systems) used pre-stored cooling capacity (e.g., phase change materials, chemical refrigerants) for localized conductive cooling, such as various cooling vests (e.g., phase change material vests, chemically activated vests, ice pack vests). (3) Control groups received passive cooling, defined as moving participants to a cool/indoor environment, removing excess clothing, and resting without external physical interventions (e.g., fanning, water spraying, ice packs). (4) Outcome measures were classified as primary or secondary. The primary outcome was mean Tc cooling rate (°C/min). Secondary outcomes included time to achieve a 1 °C reduction in Tc, total cooling magnitude, heart rate recovery rate, and adverse events (e.g., shivering, cutaneous discomfort). (5) Study designs were restricted to randomized crossover trials or parallel-group randomized controlled trials (RCTs).

### 2.3 Study selection

EndNote 21 (version 21) was used for reference management. Screening proceeded in three stages: (1) Removal of duplicate records; (2) Initial screening by two independent investigators through title and abstract evaluation; (3) Comprehensive full-text assessment of potentially eligible articles. Any discrepancies between reviewers were resolved through consensus discussion or, when necessary, arbitration by a third researcher.

### 2.4 Data extraction and transformation

Two independent investigators systematically extracted relevant information, encompassing authorship, publication year, study design, participant demographics, induction protocols, intervention specifics for both experimental and control groups, and outcome parameters. Data were compiled in Excel. Pre- and post-intervention means, standard deviations (SD), and sample sizes were extracted. For graphical data, WebPlotDigitizer 4.51 software was used (21). For studies reporting solely 95% confidence intervals (CI), appropriate statistical conversions were performed to derive SD values (22):


$$\begin{array}{l} SD=\sqrt{N}\frac{CI_{high}-CI_{low}}{2t} \end{array}$$ (1)

N represents the sample size, CI_high_ denotes the upper bound of the confidence interval, CI_low_ signifies the lower bound of the confidence interval, and t corresponds to the t-distribution (Equation 1).

When the SE is reported, it is converted to SD (Equation 2) (22).


$$\begin{array}{l} SD=\sqrt{N}\times SE \end{array}$$ (2)

For missing data, corresponding authors were contacted. Disagreements were resolved by discussion or by a third researcher. All extracted data were compiled in a standardized Excel template.

### 2.5 Study risk of bias assessment

Given that the studies anticipated for inclusion in this systematic review are predominantly randomized crossover trials, the methodological quality of each study was rigorously assessed using the Cochrane-recommended Risk of Bias tool (ROB 2 for crossover trials) (23). RevMan 5.4 was utilized to generate graphical representations of potential intra-study and inter-study biases pertaining to randomization. Two independent investigators performed the assessments, disagreements were resolved by consulting a third investigator.

### 2.6 Statistical analysis

Because most included studies used crossover designs, we used an approach for paired data. Correlation coefficients (r) were extracted from the original studies when available; *r* = 0.5 was conservatively assumed based on the Cochrane Handbook (22). Utilizing the “Generic Inverse Variance” method in RevMan 5.4, we input the mean difference (MD), SD, and sample size (N) of paired measurements, along with the specified correlation coefficient, to derive the appropriate SE.

Statistical significance was defined as a two-tailed *p* < 0.05. Heterogeneity was assessed using the *I*^2^ statistic, with values >50% indicating substantial heterogeneity (24, 25). To explore potential sources of heterogeneity, we conducted pre-specified subgroup analyses based on study protocol, stratifying primarily by Asmara (1) cooling device type (Type A: external refrigerant-based systems; Type B: wearable pre-cooling systems) and (2) ambient temperature (cooling period >30 °C vs. ≤ 30 °C). Subgroup differences were formally tested to determine whether these variables significantly modified treatment effects. For outcomes with ≥10 included studies, publication bias was assessed via funnel plots (26) and Egger's regression test (27). For outcomes with fewer than 10 studies, sensitivity analyses using the leave-one-out method were performed to evaluate result robustness (28). All statistical tests employed a significance threshold of *p* < 0.05.

## 3 Results

### 3.1 Study selection

**Figure 1:**
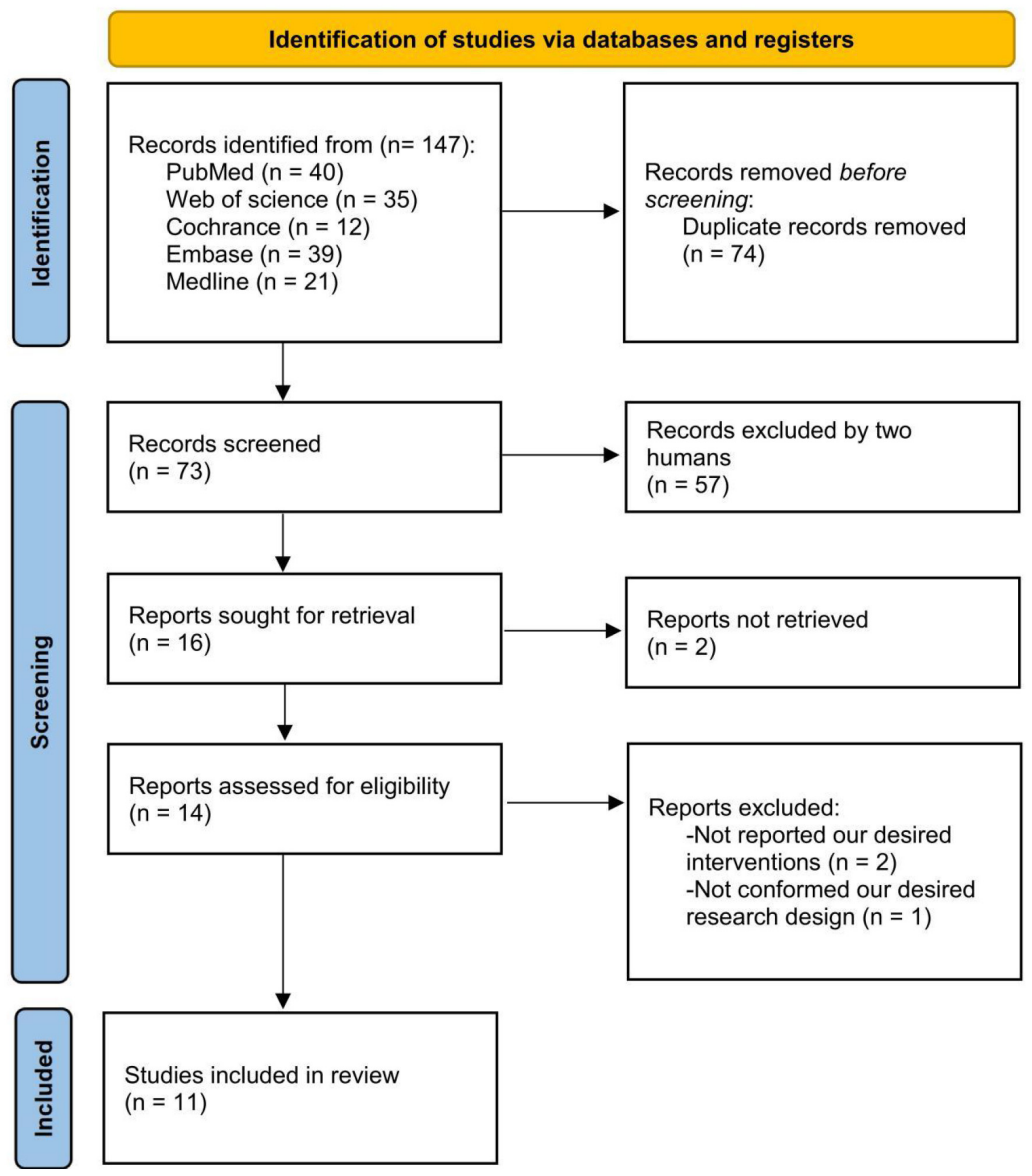
Literature screening flow chart.

Our systematic search of PubMed, Web of Science, Cochrane Library, Embase, and MEDLINE yielded 147 articles. After removing 74 duplicates, 73 articles were screened by title and abstract. 16 articles were selected for full-text assessment, of which 5 were excluded (2 due to unavailability of full texts, 1 for non-conformity with the study design, and 2 for inconsistency with the intervention criteria). The final analysis incorporated 11 eligible studies. The complete selection procedure is illustrated in Figure 1.

### 3.2 Characteristics of the studies included

This systematic review incorporated 11 original research articles published between 2008 and 2025. All studies were conducted under controlled laboratory or outdoor conditions and compared portable active cooling with passive cooling for reducing Tc in individuals with exercise-induced hyperthermia. The inclusion of both highly standardized laboratory studies and more variable outdoor field conditions introduces considerable potential heterogeneity, which should be considered when interpreting the pooled results. The included studies comprised one RCT (29) and ten randomized crossover trials (30–39); the crossover design reduces inter-individual variability and improvs statistical power.

The demographic and methodological characteristics of the included studies are presented in Table 1. The total study population included 148 participants (115 males [77.7%] and 33 females [22.3%]), predominantly healthy, physically active young adults. Sample sizes ranged from 10 to 16 participants per study, with mean ages from 21.3–31.1 years. Hyperthermia was induced by standardized exercise protocols under controlled thermal stress (high temperature and humidity). Baseline Tc ranged from 38.0 °C to 39.5 °C, representing moderate to severe hyperthermia. Notably, no participants exhibited central nervous system dysfunction (e.g., disorientation, seizures, or coma), and only a minority reached Tc ≥40.0 °C. Thus, the included studies pertain to exercise-induced hyperthermia rather than clinical exertional heat stroke; this distinction is addressed in the discussion limitations. Additionally, participants using type B devices had lower pre-intervention core temperatures (38.4–38.9 °C) compared with those in type A studies (38.8–39.5 °C), which may have facilitated faster passive cooling and attenuated the apparent treatment effect of type B devices.

**Table 1:** Essential characteristics of the included literature.

Study	Study design	Participant	Included core body temperature criteria and induction methods	Intervention	Control	Methodologies for core temperature measurement	Primary outcome measures (cooling rate, °C/min) (mean ±SD)
Lopez RM et al. (29)	RCT	*N* = 10, Men Ration = 100% Age = 25.6 ± 1.6 years Weight = 80.3 ± 13.7 kg Healthy male participants without any documented history of febrile illnesses or chronic medical conditions.	The treadmill exercise protocol elicited a core body temperature elevation to 38.7 ± 0.3 °C. The experimental conditions involved high ambient temperature (33.1 ± 3.1 °C) and relative humidity (55.1 ± 8.9%), with exercise termination criteria set at a 3.27 ± 0.1% reduction in body weight.	cooling vests	Post-exercise recovery occurred under thermoneutral conditions (26.6 ± 2.2 °C, 55.4 ± 5.8% relative humidity).	Rectal thermistor	Cooling vests group: 0.0298 ± 0.0072 control group: 0.0280 ± 0.0074^ns^
Brade C et al. (31)	Randomized crossover trial	*N* = 12, Men Ratio*n* = 100% Age = 21.3 ± 1.1 years Height = 182.7 ± 7.1 cm weight = 76.2 ± 9.5 kg healthy male participants engaged in physical activity.	Hyperthermia was naturally induced (mean peak core temperature reaching approximately 38.5 °C) through a 30 min cycling protocol performed at 75% of VO_2_max intensity under controlled high-temperature conditions (35.0 ± 1.4 °C, relative humidity 52 ± 4%)	PC17 cooling vests	Following exercise, participants recovered in a thermoneutral environment (24.9 ± 1.8 °C, relative humidity 39 ± 10%).	Ingestible capsule thermometer	PC17 cooling vests: 0.038 ± 0.007^*^ Control group: 0.034 ± 0.010^ns^
				Gel cooling vests			Gel cooling vests: 0.040 ± 0.009^*^ Control group: 0.034 ± 0.010^ns^
Hostler D et al. (30)	Randomized crossover trial	*N* = 18, Men Ratio*n* = 77.78% age = 31.1 ± 7.6/25.5 ± 5.2 years Height = 176. 2 ± 5.5/157.7 ± 2.9 cm Weight = 80.2 ± 12.2/58.5 ± 6.7 kg Healthy individuals without any history of cardiovascular or respiratory diseases.	Core body temperature was elevated to >38.0 °C through exercise while wearing protective clothing on a treadmill in an environmental chamber maintained at 35.1 ± 2.7 °C.	Water circulation cooling vests	Baseline measurements were obtained under resting conditions at an ambient temperature of 24.0 ± 1.4 °C.	Ingestible capsule thermometer	Water circulation cooling vests: 0.041 ± 0.022 Control vests: 0.047 ± 0.031^ns^
DeMartini JK et al. (32)	Randomized crossover trial	*N* = 16, Men Ration = 56.25% Age = 24 ± 6 years^**^ Height = 182 ± 7 cm^**^ Weight = 74.03 ± 9.17 kg^**^ The subject was in good health with no history of chronic diseases and had been afebrile for the preceding 3 years.	Post-exercise rectal temperature (Tre) was recorded as 38.73 ± 0.12 °C Participants engaged in outdoor ball sports (including soccer and ultimate frisbee) for 45–60 min under warm environmental conditions (mean wet-bulb globe temperature [WBGT]: 26.64 ± 4.71 °C).	Game Ready Active Cooling Vest™	Following exercise, subjects rested on an unshaded bench adjacent to the sports field (WBGT: 26.30 ± 4.13 °C).	Rectal thermistor, 10 cm posterior to the anal sphincter insertion point.	GRV group: 0.040 ± 0.015 Control group: 0.042 ± 0.015^ns^
				Nike ice vest™			NIV group: 0.050 ± 0.015 Control group: 0.042 ± 0.015^ns^
Luhring KE et al. (33)	Randomized crossover trial	*N* = 16, Men Ration = 56.25% Age = 26 ± 4.7 years^**^ Height = 176 ± 9 cm^**^ Weight = 72.5 ± 9.0 kg^**^ The subject exhibited no signs of illness and had no prior history of febrile episodes.	Under elevated ambient conditions (33.4 °C ± 0.8 °C, relative humidity 55.7% ± 1.9%), autonomous thermoregulatory behavior was observed when core body temperature (rectally measured) reached ≥39.0 °C.	Tarp-assisted cooling with oscillation (TACO)	The subject subsequently assumed a resting posture on a dry, waterproof surface.	Rectal thermistor	TACO group: 0.14 ± 0.06 Control group: 0.04 ± 0.02^#^
Butts CL et al. (34)	Randomized crossover trial	*N* = 13, men ratio *n* = 84.62% Age = 23 ± 3 years^**^ Height = 176.5 ± 10.3 cm^**^ Weight = 78.6 ± 15.3 kg^**^ The participants were in good physical condition with no chronic diseases and no history of febrile illness within the preceding 3 years.	Tre was monitored and maintained at ≥39.0 °C during exercise, achieved through running and sprinting in a high-temperature, high-humidity environment (34.4 ± 1.4 °C, relative humidity 54.4 ± 4.1%).	Ice-sheet cooling (ISC)	Following exercise, participants rested in the shade of an outdoor sports field (ambient temperature ~34.4 °C, relative humidity 54.4%) without any active cooling interventions.	Rectal thermistor	ISC group: 0.06 ± 0.02 Control group: 0.05 ± 0.02^ns^
Hosokawa Y et al. (35)	Randomized crossover trial	*N* = 14, men ratio *n* = 57.14% Age = 25 ± 4/22 ± 2 years Height = 181.1 ± 7.4/163.5 ± 6.7 cm Weight = 86.7 ± 10.5/61.3 ± 6.7 kg Health and recreational activities.	Participants performed treadmill running in a controlled high-temperature environment (39.5 ± 3.1 °C, 38.1 ± 6.7% relative humidity) until reaching either a core Tre of ≥39.5 °C or volitional exhaustion.	Tarp-assisted method utilizing a waterproof fabric combined with ice water immersion	For comparison, a separate sedentary condition was conducted in an environmental chamber maintained at identical thermal parameters (39.5 °C ambient temperature, 38.1% relative humidity).	Rectal thermistor, 10 cm posterior to the anal sphincter insertion point.	Trap group: 0.17 ± 0.07 Contril group: 0.04 ± 0.01^#^
Hosokawa Y et al. (36)	Randomized crossover trial	*N* = 14, men ratio *n* = 57.14% Age = 25 ± 4/22 ± 2 years Height = 181.1 ± 7.4/163.5 ± 6.7 cm Weight = 86.7 ± 10.5/61.3 ± 6.7 kg The subjects were healthy individuals with no history of febrile illness.	Exercise was performed on a treadmill under high-temperature conditions until the target core body temperature was achieved (Tre ≥39.0 °C, 39.8 ± 1.9 °C; relative humidity: 37.4 ± 6.9%).	CAERvest^®^ Chemical Activation Cooling vest	Following the intervention, participants remained seated at rest without active cooling measures.	Rectal thermistor, 10 cm posterior to the anal sphincter insertion point.	VEST group: 0.06 ± 0.02 Control group: 0.04 ± 0.01^ns^
Caldwell AR et al. (37)	Randomized crossover trial	*N* = 11, men ratio *n* = 90.91%^***^ Age = 24.3 ± 6.2 years^**^ Height = 177.6 ± 8.8 cm^**^ Weight = 84.3 ± 15.8 kg^**^ Healthy and physically fit.	Participants were required to walk in a controlled high-temperature environment (40 °C, 30% relative humidity) until their gastrointestinal temperature reached ≥38.0 °C, with the protocol terminating either when core body temperature exceeded ≥39.2 °C or after a maximum duration of 3 hours.	Ice-sheet cooling (ISC)	Following the walking phase, subjects assumed a supine position for recovery without any active cooling interventions.	Gastrointestinal telemetry pill	ISC group: 0.068 ± 0.0195 Control group: 0.047 ± 0.0130^#^
Wang X et al. (38)	Randomized crossover trial	*N* = 12, men ratio *n* = 100% Age = 22 ± 1.3 years Height = 176 ± 8 cm Weight = 66.3 ± 4.2 kg healthy, trained	Participants were subjected to exercise-induced hyperthermia (mean core temperature 39.41 °C) by performing 30 min of endurance running in a humid-heat environment (30 °C, 66% relative humidity).	PCM hypothermic blanket	Resting in a static position under controlled conditions (25 °C, 66% relative humidity, 1.2 m/s wind speed).	Ingestible capsule thermometer	PCM group: 0.12 ± 0.02 Control group: 0.08 ± 0.02^#^
Li X et al. (39)	Randomized crossover trial	*N* = 12, men ratio *n* = 100% age = 22 ± 1.6 years health, endurance training	Exercise-induced hyperthermia (mean core temperature >39.0 °C) was induced under controlled environmental conditions (40.0 ± 0.3 °C ambient temperature, 40 ± 3% relative humidity, and no air movement) through a 3-km running protocol in a thermoneutral chamber.	water-circulating cooling blanket	Participants maintain a supine position on a wooden platform. The cooling environment was maintained at approximately 31 °C with a relative humidity of 65%.	Rectal thermistor	Round 1: LWC group: 0.0280 ± 0.0261 Control group: 0.0167 ± 0.0261^ns^
							Round 2: LWC group: 0.0380 ± 0.0293Control group: 0.0227 ± 0.0481^#^

^*^The experimental data represent the mean cooling rate calculated from peak body temperature (recorded either at the cessation of exercise or 5min post-recovery initiation) to the conclusion of the recovery period (60min).

^**^Demographic characteristics (age and height) of the study participants were reported as aggregate means without sex-specific stratification.

^***^The participant count reflects the final cohort that successfully completed the full experimental protocol.

^#^Statistically significant difference between the intervention and control groups (*p* < 0.05) as reported in the original study.

^ns^Indicates non-significant difference.

Cooling interventions were classified into two categories: type A devices (six studies) included waterproof canvas-assisted cooling (*n* = 2), ice bed sheet cooling (*n* = 2), and cooling blankets (*n* = 2); type B devices (seven studies) included phase-change material vests, chemically activated vests, water-circulating vests/blankets, and ice pack vests. Control groups uniformly used passive cooling (removal to a designated rest environment), although ambient temperatures varied substantially (24.0 °C to exercise-matched conditions), which may introduce heterogeneity. Tc was monitored using rectal probes (seven studies) or ingestible capsule thermometers (four studies). All studies reported the primary outcome—mean Tc cooling rate—with standardized measurement protocols.

### 3.3 Risk of bias

**Figure 2:**
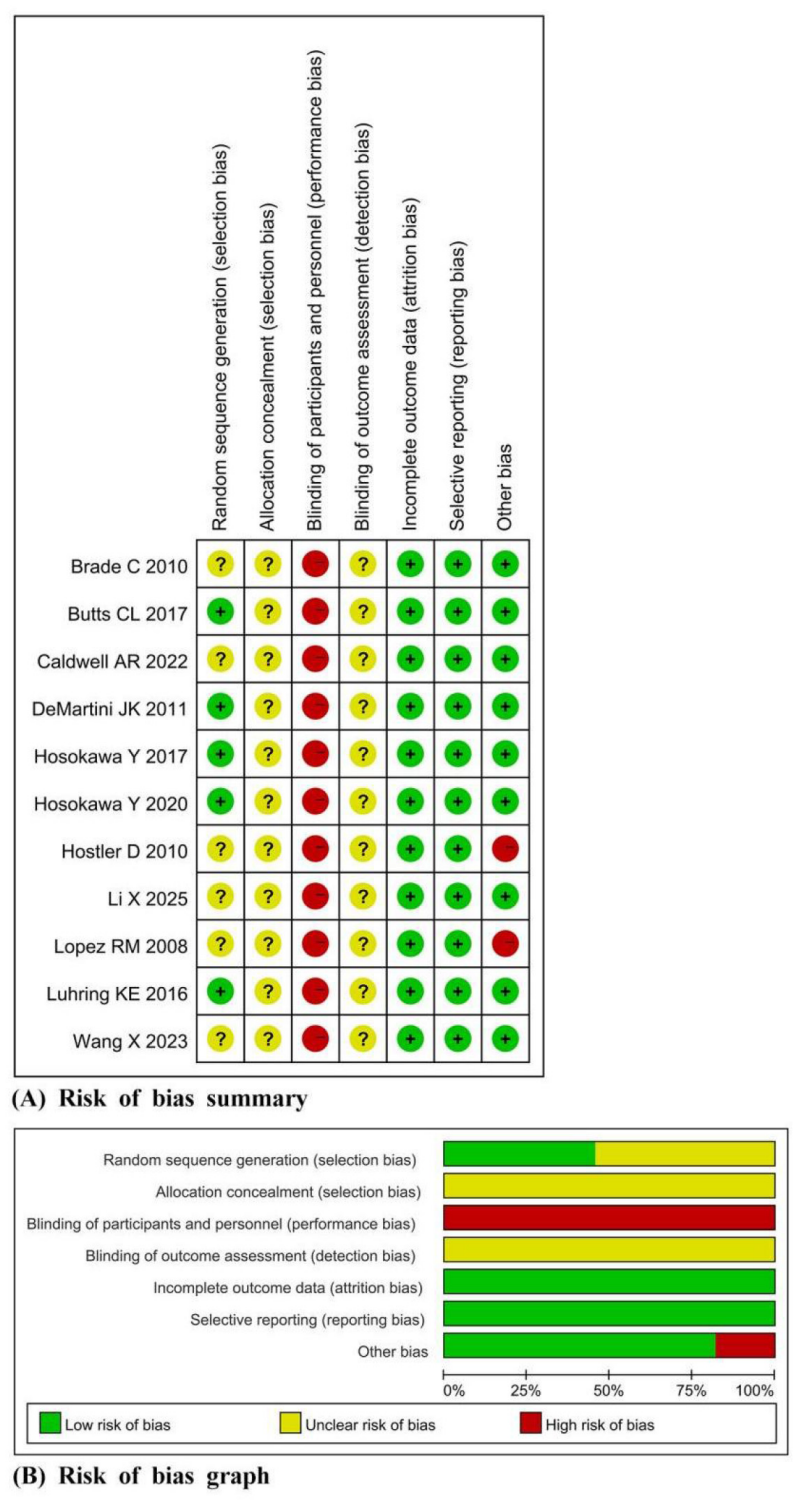
Risk of bias assessment of included studies.

The risk of bias was accessed using the RoB 2 tool for 11 included studies, with detailed results illustrated in Figure 2. The primary source of bias was lack of blinding of participants and personnel; all studies received a “high risk” rating in this domain. This limitation is inherent to EHS cooling research and is addressed in the discussion.

For other domains, five studies showed “high risk” for random sequence generation, and six were rated “unknown risk” due to insufficient methodological details. Allocation concealment and blinding of outcome assessment were poorly reported across all studies, resulting in “unknown risk” for both criteria. All studies had “low risk” for incomplete outcome data and selective reporting. For other potential biases, nine studies were rated “low risk,” whereas two remained “unknown risk” due to limited reporting.

### 3.4 Effect size of the intervention

#### 3.4.1 Cooling rate

**Figure 3:**
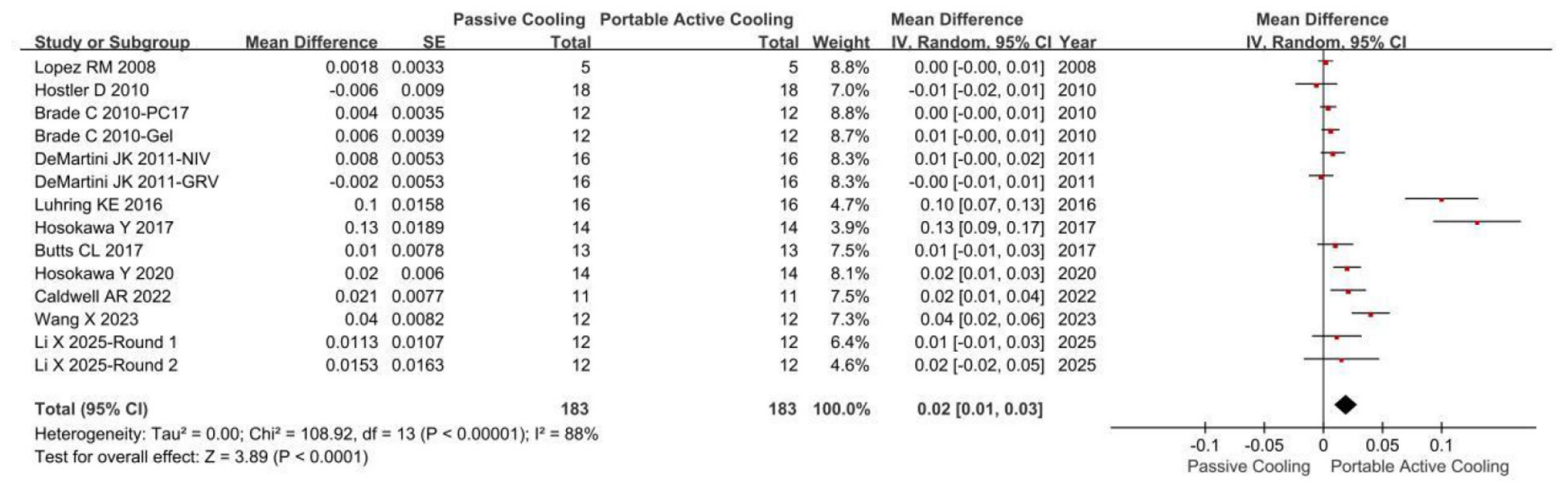
Forest plot demonstrating pooled results for Cooling rate.

To compare the efficacy of portable active cooling vs, passive cooling on Tc cooling rates in individuals with exercise-induced hyperthermia, we performed a meta-analysis of 11 RCTs (including crossover designs). A random-effects model was used to account for expected heterogeneity across intervention protocols, experimental conditions, and participant characteristics. The pooled analysis showed that portable active cooling produced a significantly higher mean cooling rate (0.02 °C/min, 95% CI: 0.01–0.03) than passive cooling (Figure 3). This difference was statistically significant (*p* < 0.00001), with the forest plot clearly favoring portable active cooling. However, substantial heterogeneity was observed (*I*^2^ = 88%), indicating variations in study design, intervention implementation, or measurement methodologies—rather than random chance—likely contributed to the disparate findings. To elucidate potential sources of heterogeneity, a prespecified subgroup analysis was conducted.

##### 3.4.1.1 Subgroup analysis: by device type

**Figure 4:**
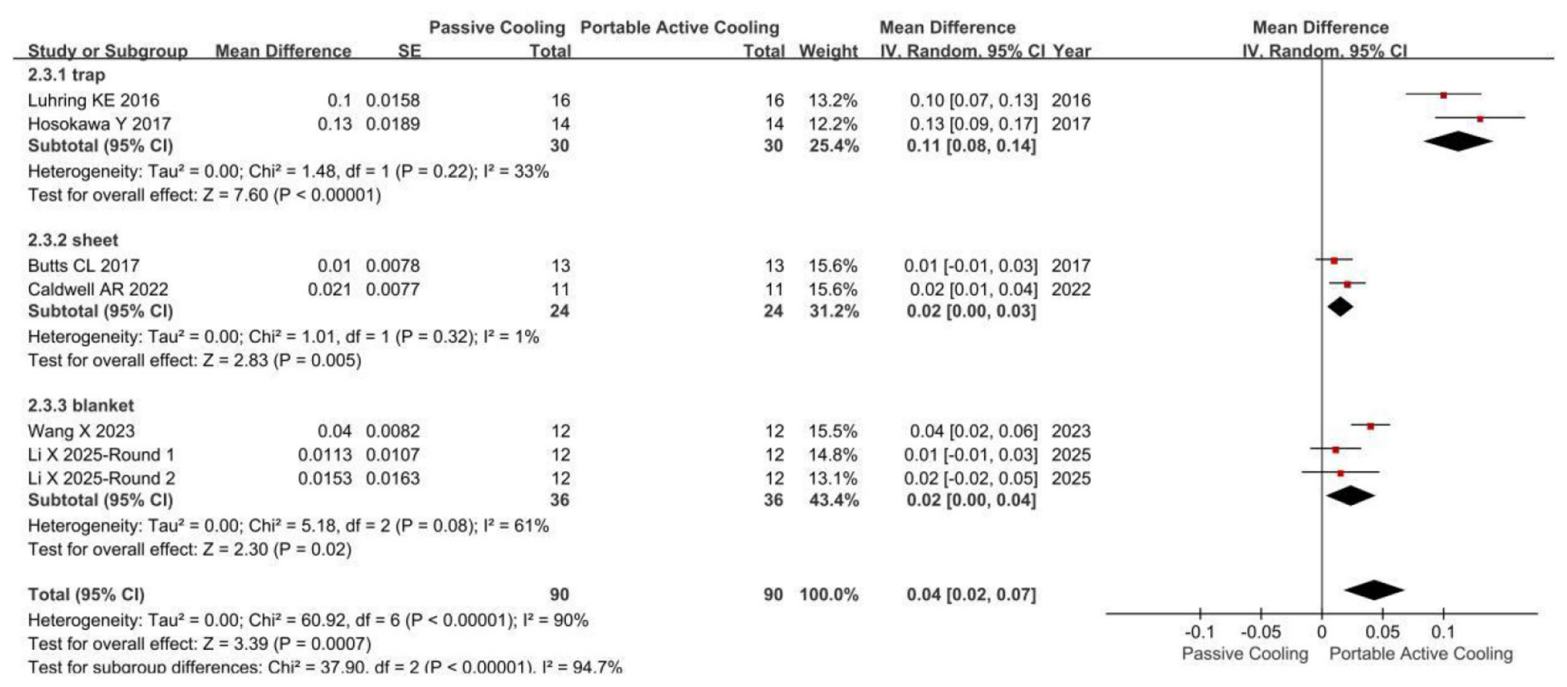
Forest plot of cooling rates for external-coolant based active cooling devices (Type A) vs. passive cooling.

**Figure 5:**
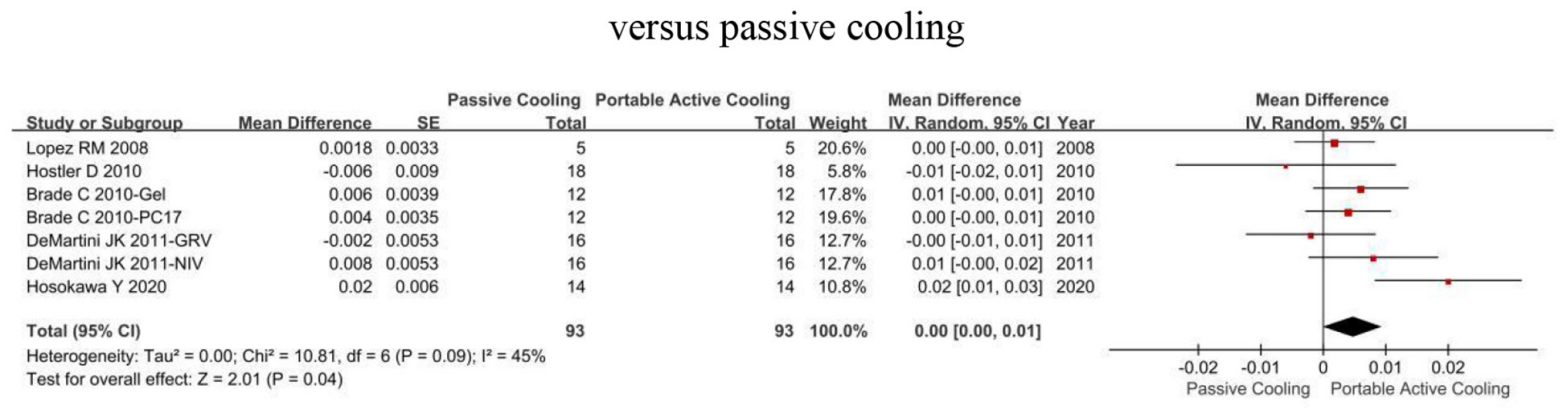
Forest plot of cooling rates for cooling vests (Type B) vs. passive cooling.

To examine the sources of heterogeneity and compare efficacy across portable active cooling devices, we stratified the intervention into type A (external refrigerant-based systems) and type B (wearable pre-cooling systems). Type A use externally supplied cooling media (e.g., ice or water) to achieve conductive and/or evaporative cooling over large body surfaces. Evaporative heat loss is enhanced when ice-water-soaked waterproof fabrics or sheets are applied to the skin: the cold liquid continuously wets the surface, and as it evaporates (even in humid environments, facilitated by the temperature gradient and renewal of the liquid film), substantial heat is extracted from the body. Six studies were included in this category, further classified into three subtypes. (1) Waterproof fabric-assisted cooling (2 studies) yield a pooled cooling rate of 0.11 °C/min (95% CI: 0.08–0.14, *p* < 0.00001, *I*^2^ = 33%). (2) Ice sheet cooling (two studies) showed a pooled cooling rate of 0.02 °C/min (95% CI: 0.00–0.03, *p* = 0.005, *I*^2^ = 1%). (3) Circulating cooling blanket (two studies) had a pooled cooling rate of 0.02 °C/min (95% CI: 0.00–0.04, *p* = 0.02, *I*^2^ = 61%). Collectively, type A devices demonstrated significant cooling efficacy compared with passive cooling (pooled MD = 0.04 °C/min; 95% CI: 0.02–0.07; *p* = 0.0007), but heterogeneity remained high (*I*^2^ = 90%), as shown in Figure 4. In contrast, type B devices (seven studies) showed a pooled effect size of 0.00 °C/min (95% CI: 0.00–0.01, *p* = 0.09) with moderate heteroheneity (*I*^2^ = 45%), indicating no statistically significant advantage over passive cooling (Figure 5). These findings suggest that device type is the primary moderator of heterogeneity, and the evaporative mechanism of type A devices likely contributes to their superior cooling efficacy, particularly in warm environments.

##### 3.4.1.2 Subgroup analysis: by ambient temperature during the cooling period

**Figure 6:**
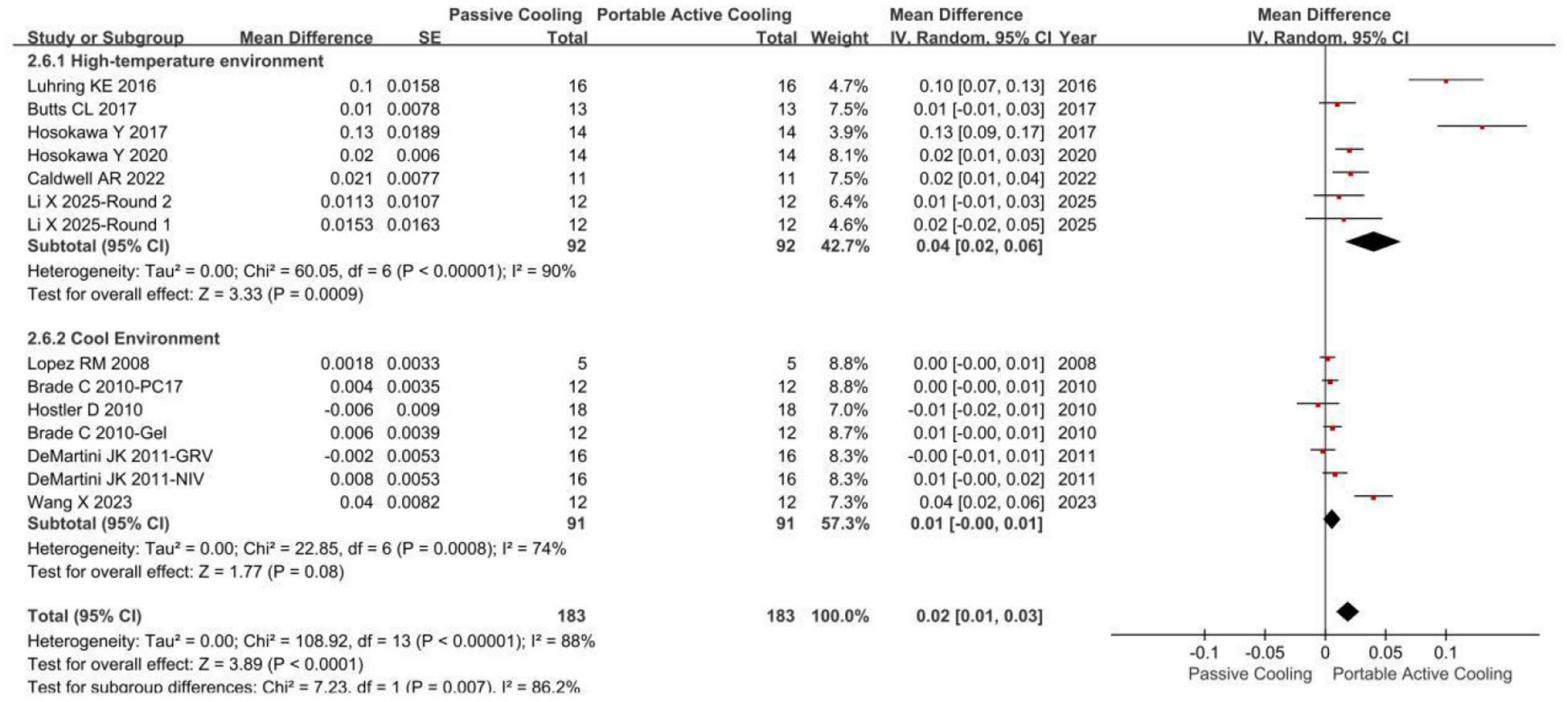
Forest plot of cooling rates for portable active cooling vs. passive cooling, stratified by ambient temperature during the cooling phase.

We also stratified studies by ambient temperature during the cooling phase: high-temperature environment (>30 °C) vs. cool environment ( ≤ 30 °C). The analysis (Figure 6) showed that ambient temperature significantly modified cooling efficacy (between-subgroup difference test, *p* = 0.007). In high-temperature environments, portable active cooling was superior to passive cooling (MD = 0.04 °C/min, 95% CI: 0.02–0.06, *p* = 0.0009), but heterogeneity (*I*^2^ = 90%). In cool environments, no significant difference was observed (MD = 0.01 °C/min, 95% CI: – 0.00–0.01, *p* = 0.08), with moderate heterogeneity (*I*^2^ = 74%). Further stratified analysis by both device type and ambient temperature (Table 2) confirmed that device classification was the principal determinant of cooling efficacy. Type A devices showed a significant cooling advantage in high-temperature environments (pooled MD = 0.05 °C/min, 95% CI: 0.01–0.08, *p* = 0.005, *I*^2^ = 92%), whereas type B devices did not show a significant benefit in cool environments (pooled MD = 0.00 °C/min, 95% CI: −0.00–0.01, *p* = 0.07, *I*^2^ = 0%). Limited evidence was available for type A in cool environments (one study) and type B in high-temperature environments (one study), precluding firm conclusions for those combinations.

**Table 2:** Subgroup analysis of cooling rates stratified by device type and ambient temperature.

Device type	Ambient temperature	Number of studies	Direction of effect	Qualitative conclusion	Included in the study
Type A	High-temperature environment	5	Significant positive effect pooled MD = 0.05, 95% CI: 0.01–0.08, *p* = 0.005, *I*^2^ = 92%	Under the target scenario (high temperature), Type A equipment is clearly effective.	(33–35, 37, 39)
	Cool environment	1	Significant positive effect single study MD = 0.04, 95% CI: 0.02 to 0.06, *p* < 0.00001	There is a lack of evidence to support the advantages of Type A devices in cool environments.	(38)
Type B	High-temperature environment	1	Significant positive effect single study MD = 0.02, 95% CI: 0.01–0.03, *p* = 0.0009	In the target scenario (high temperature), the evidence of the performance of Type B devices is extremely limited.	(36)
	Cool environment	4	No significant effect Pooled MD = 0.00, 95% CI: −0.00–0.01, *p* = 0.07, *I*^2^ = 0%	In a cool environment, Type B devices are completely ineffective	(29–32)

#### 3.4.2 Descriptive synthesis of secondary outcomes

This systematic review performed a descriptive meta-analysis of key secondary outcomes, in addition to the primary outcome (core body temperature cooling rate), to comprehensively assess intervention efficacy across multiple dimensions, including cooling efficiency, physiological recovery, and safety (Table 3). For type A devices, three studies reported substantially shorter cooling times to achieve a 1 °C reduction (approximately 5.9–9.3 min) compared with passive cooling (approximately 25–26.6 min). In contrast, most studies on type B devices did not find statistically significant reductions in cooling time. Regarding total cooling magnitude, type A devices consistently achieved greater temperature reductions within fixed observation periods (e.g., first 3 or 30 min) than passive cooling. Multiple studies on type B devices reported no significant differences in total cooling magnitude compared with passive cooling. Evidence on heart rate recovery was limited; one study on a phase-change material blanket (type A) reported faster recovery of heart rate and heart rate variability compared with passive cooling. For adverse events, cold-induced shivering was relatively common with type A devices, with one study reporting an incidence of 50%. Local discomfort from cold saline infusion was also noted. Serious adverse events were rare, but safety reporting across studies was incomplete, precluding comparative assessments.

**Table 3:** Descriptive summary of secondary outcomes.

Secondary outcomes category	Type A main findings	Type B main findings	Summary
Time required for core body temperature to drop by 1 °C	**The cooling duration was markedly reduced compared to passive cooling approaches**. 1. Luhring et al. (33) reported that TACO achieved a cooling time of approximately 7.14 min, whereas passive cooling required approximately 25 min. 2. Hosokawa et al. (35) observed that the tarp method reduced cooling time to approximately 5.88 min, compared to 25 min for passive cooling. 3. Caldwell et al. (37) further demonstrated that the median time to reach 38 °C (intra-scrotal cooling) was 9.3 min for active cooling, vs. 26.6 min for passive cooling.	**The existing evidence remains limited and inconsistent, with most studies failing to demonstrate a statistically significant advantage**. 1. Lopez et al. (29) found no significant difference in time to baseline recovery (43.8 vs. 56.6 min). 2. Hosokawa et al. (36) reported a shorter estimated cooling time (approximately 16.7 min for the cooling vest vs. 25 min for passive cooling), the study had a small sample size and was conducted in a specialized environment. 3. Brade et al. (31) and DeMartini et al. (32) did not observe a statistically significant reduction in cooling time.	Type A devices exhibit a distinct superiority over passive cooling in achieving rapid critical temperature thresholds, whereas the effectiveness of type B devices has not been consistently substantiated in the literature.
Overall temperature drop	**During the specified observation period, active cooling demonstrated superior efficacy compared to passive cooling**. 1. Butts et al. (34) reported a core temperature reduction of 0.33 °C within the initial 3 min with active cooling vs. 0.03 °C with passive cooling. 2. Wang et al. (38) observed significantly greater cooling amplitude using PCM blankets compared to natural cooling over 30 min.	Multiple studies Lopez et al. (29), Brade et al. (31), Hostler et al. (30), DeMartini et al. (32) et al. found no statistically significant difference in total cooling magnitude between Class B devices and passive cooling methods.	Type A devices achieved greater absolute cooling within shorter timeframes, whereas types B devices showed no significant additional benefit in overall cooling magnitude.
Heart rate recovery rate	Wang et al. (38) noted that patients treated with PCM cooling blankets exhibited significantly accelerated recovery of heart rate and heart rate variability compared to the passive cooling group.	Not reported as a primary outcome system in type B device studies	Preliminary evidence suggests that effective active cooling may promote cardiovascular recovery, but more research is needed to confirm this.
Adverse event	Chills are relatively common. 1. Luhring et al. (33) and Caldwell et al. (37) documented chills during ice/ice water application, with Caldwell's study reporting an incidence of 50%. 2. Hostler et al. (30) observed upper limb discomfort associated with cold saline infusion.	Serious adverse events are rare, but safety reports are generally incomplete	Type A methods exhibited a higher risk of chills; overall, severe adverse events were infrequent, but inconsistent monitoring and reporting across studies limited comparative safety assessments.
Summary	It shows clear advantages in the rate, duration, and extent of cooling, but is accompanied by a higher risk of shivering.	Based on the current evidence, it has not been consistently proven to be significantly more effective in cooling than passive cooling.	Type A equipment is suitable for on-site emergency care that requires rapid cooling; type B equipment's effectiveness as an auxiliary or maintenance measure needs further evaluation. Safety monitoring needs to be standardized.

### 3.5 Sensitivity analysis

We conducted sensitivity analyses to assess the robustness of the primary outcome findings. Leave-one-out showed that no single study dispriportionately influenced the pooled effect; the MD ranged from 0.01 to 0.03 °C/min, and all *p* < 0.05. A fixed-effects model producted similar results (MD = 0.01 °C/min, 95% CI: 0.01–0.01, *p* < 0.00001) compared with the primary random-effects model (MD = 0.02 °C/min, 95% CI: 0.01–0.03, *p* < 0.00001). In subgroup analyses, excluding Hosokawa Y's (2020) study form the type B device analysis reduced heterogeneity from 45 to 0% without materially altering the MD, CI, or p-value.

**Figure 7:**
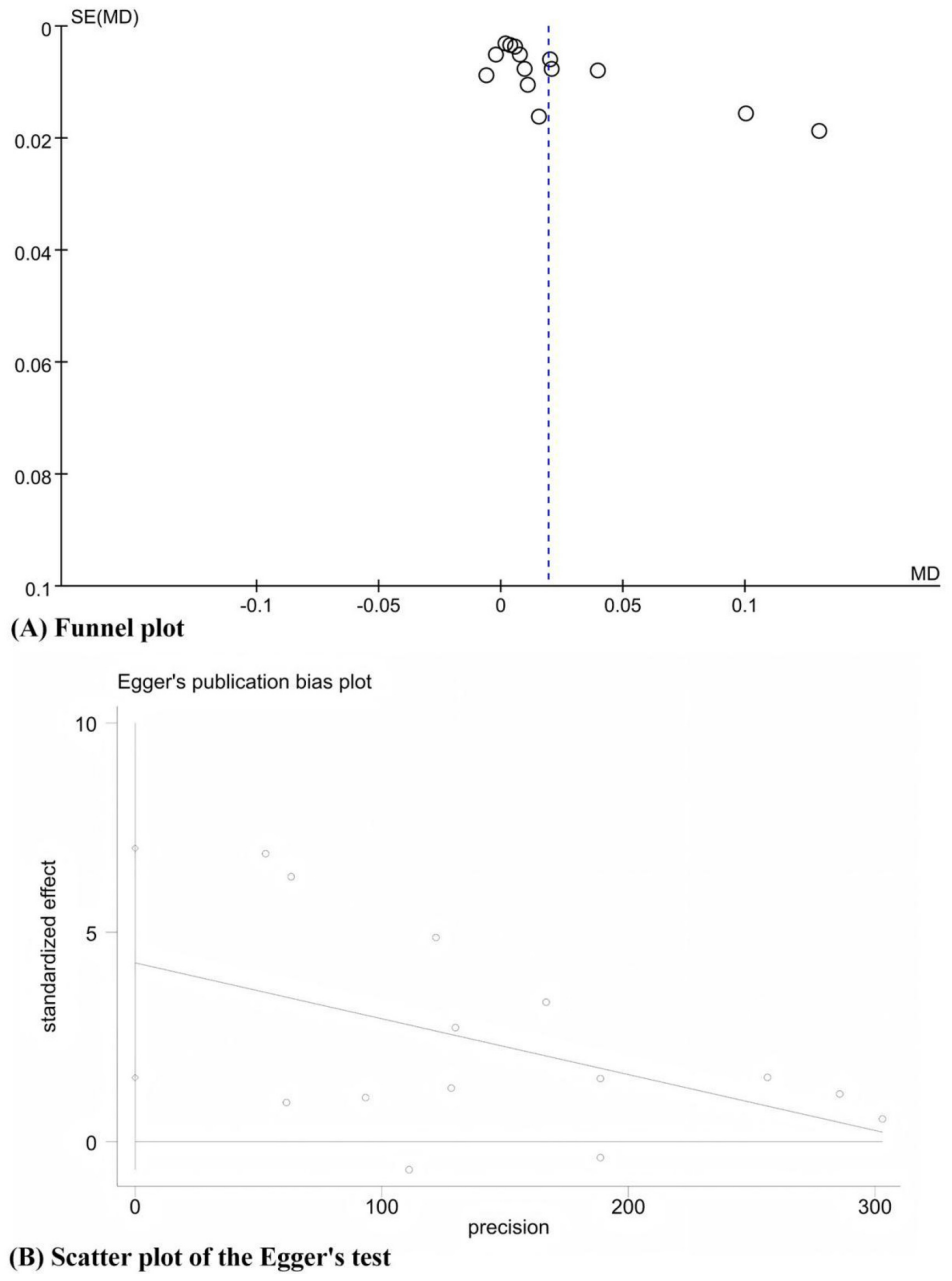
Assessment of publication bias for the primary outcome (core temperature cooling rate).

Publication bias was assessed using funnel plots and Egger's test because the primary outcome included ≥10 studies (Figure 7). Visual asymmetry in funnel plots and a significant Egger's test (*t* = 3.40, *p* = 0.005) suggested potential publication bias or small-study effects. Although high heterogeneity (*I*^2^ = 88%) may contribute to funnel plot asymmetry, the significant Egger's test indicates that the distribution of effect sizes is not fully explained by heterogeneity alone. This suggests that negative or null results may be under-represented in small studies, potentially inflating the effect estimates for portable active cooling interventions. This potential bias warrants careful consideration when interpreting and generalizing the meta-analytic results.

## 4 Discussion

This study systematically reviewed and meta-analyzed the comparative efficacy of portable active cooling devices vs. passive cooling to reduce Tc among patients with EHS or hyperthermia. The main findings are as follows: first, portable active cooling devices produced a significantly higher cooling rate than passive cooling, although substantial heterogeneity was present. Second, device type was the primary moderator of this heterogeneity. Type A devices consistently showed superior cooling efficacy, whereas type B devices did not demonstrate a statistically significant advantage over passive cooling. Third, ambient temperature modified the effect, but device type remained the predominant determinant. In high-temperature environments (>30 °C), which are the most relevant for EHS management, the cooling advantage of type A devices were particularly pronounced.

Although the pooled MD of 0.02 °C/min reached statistical significance, its clinical relevance warrants careful interpretation. Given that most included studies involved individuals with mild-to-moderate hyperthermia (Tc < 40 °C) rather than full-blown exertional heat stroke, the clinical benefit of such a small difference in cooling rate remains uncertain, particularly when weighed against device cost, availability, and potential adverse effects such as shivering. For the majority of type B devices, no clinically meaningful advantage was observed.

Regardless of device selection, the first and most basic step in field management of exertional heat stroke should be immediate removal of the patient from the hot environment to a shaded or air-conditioned area (40, 41). Passive cooling alone (moving to a cool environment, removing clothing, and resting) remains the fundamental initial intervention, as it reduces ongoing environmental heat gain and facilitates endogenous heat dissipation (2). Our subgroup analysis showed that in cool environments ( ≤ 30 °C), the additional benefit of portable active cooling over passive cooling was not statistically significant, underscoring the critical importance of environmental control before or alongside device-based cooling. A systematic review of passive rest cooling rates found that passive rest alone often fails to reverse elevated core temperatures in a timely manner, particularly in hot environments (42), further supporting the necessity of aggressive active cooling when environmental conditions are unfavorable.

From a pathophysiological perspective, EHS is not merely a consequence of elevated Tc but a systemic crisis triggered by heat toxicity, involving profound inflammatory responses and coagulopathy (2, 5, 9). Tc >40 °C cause direct cellular damage, including membrane disruption, mitochondrial dysfunction, and intestinal barrier injury, leading to endotoxin translocation and increasing the risk of cytokine storm, endothelial injury, and DIC (5, 7, 9). Therefore, the primary goal of cooling is to rapidly reduce Tc below the critical threshold (< 39.0 °C) to mitigate tissue damage and the inflammatory cascades (14, 15). The cooling rate is directly associated with the extent of organ dysfunction and mortality (13).

Our subgroup analysis by device type revealed important differences within type A devices. Among the six type A studies, three subtypes were identified: waterproof fabric-assisted cooling (two studies) yielded a pooled cooling rate of 0.11 °C/min (95% CI: 0.08–0.14, *I*^2^ = 33%); ice sheet cooling (two studies) gave 0.02 °C/min (95% CI: 0.00–0.03, *I*^2^ = 1%); and circulating cooling blankets (two studies) gave 0.02 °C/min (95% CI: 0.00–0.04, *I*^2^ = 61%). The markedly higher cooling rate of waterproof fabric-assisted cooling (often referred to as tarp-assisted cooling with oscillation) likely results from a combination of conductive and evaporative heat exchange. When an ice-water-soaked waterproof fabric is applied to the skin, the cold liquid continuously wets the body surface. As the liquid evaporates—even in humid environments, the temperature gradient between the warm skin and the cold liquid film, together with the constant renewal of the liquid layer, sustains evaporative heat loss. This mechanism extracts substantial heat from the body, beyond what conduction alone would achieve. In contrast, ice sheet cooling (e.g., ice packs or cold-water-soaked sheets placed over the body without active wetting) relies primarily on conduction, with minimal evaporative contribution, explaining its lower cooling rate. Circulating cooling blankets also depend mainly on conduction, and their performance may vary with water temperature, flow rate, and coverage area, leading to moderate heterogeneity (*I*^2^ = 61%).

The present analysis provides compelling evidence supporting the preferential use of type A devices, particularly waterproof fabric-assisted methods, when cold-water immersion is unavailable. These devices achieve superior cooling rates and significantly reduce the time needed to lower core temperature by 1 °C (approximately 5.9–9.3 min vs. 25–26.6 min for passive cooling), which has critical implications for achieving the “golden 30-min” therapeutic window. In contrast, conventional cooling vests (type B devices) showed limited efficacy (pooled MD = 0.00 °C/min, 95% CI: 0.00–0.01, *p* = 0.09). This lack of effect is likely attributable to their dependence on finite pre-stored cooling capacity, rapid thermal equilibration under sustained heat stress, and relatively small body surface area coverage, which collectively compromise heat exchange efficiency. These findings challenge the common practice of selecting devices based primarily on portability and underscore the need to prioritize cooling mechanisms and thermodynamic performance.

Beyond these practical considerations, the lack of significant difference in cooling efficacy between type B devices and passive cooling can be attributed to multiple thermodynamic, physiological, and design limitations. First, type B devices depend on a finite cold storage capacity; phase-change materials or chemical refrigerants rapidly reach thermal saturation under high ambient temperatures, failing to sustain the cooling required during the “golden 30-min” window (43). Second, these vests primarily cover the anterior and posterior torso, an area with limited total heat exchange capacity (44, 45). A deeper physiological explanation lies in the fundamental differences in thermal exchange between glabrous (hairless) and non-glabrous skin. Glabrous skin surfaces—the palms, soles, face, and ears—constitute only a small percentage of total body surface area but contain specialized vascular structures called arteriovenous anastomoses (AVAs). These direct connections between small arteries and veins bypass the nutritive capillary beds and shunt blood to venous plexuses, allowing large volumes of blood to be brought close to the body surface for highly efficient heat exchange (46–48). In summary, the ineffectiveness of type B devices results from a combination of limited cold storage capacity, inadequate coverage area, and the physiological mismatch of targeting non-glabrous rather than glabrous skin.

The influence of ambient temperature was also examined. In high-temperature environments (>30 °C), portable active cooling was superior to passive cooling (MD = 0.04 °C/min, 95% CI: 0.02–0.06, *p* = 0.0009), whereas in cool environments ( ≤ 30 °C), the difference was not significant (MD = 0.01 °C/min, *p* = 0.08). Further stratification by both device type and ambient temperature (Table 2) confirmed that device classification is the principal determinant. Type A devices showed a significant cooling advantage in high-temperature settings (pooled MD = 0.05 °C/min, 95% CI: 0.01–0.08, *p* = 0.005), but heterogeneity remained high (*I*^2^ = 92%), suggesting additional factors such as ice-water ratio, application duration, and humidity also contribute. For type B devices in cool environments, no benefit was observed (MD = 0.00 °C/min, *I*^2^ = 0%). Limited evidence was available for type A in cool environments (one study) and type B in high-temperature environments (one study), precluding firm conclusions for those combinations. Overall, the cooling efficacy of portable active cooling systems is dictated more by the device's core cooling mechanism than by ambient temperature alone.

The observed heterogeneity in the pooled analysis (*I*^2^ = 88%) may arise not only from device type but also from variations in refrigerant parameters (e.g., water temperature, ice-water ratio), application duration, and ambient conditions (temperature, humidity, airflow velocity). This underscores the need to standardizing operational protocols (e.g., mandating ice-water mixtures rather than cold water alone) and to explicitly environmental parameters in future studies. Additionally, type A devices were associated with a higher incidence of cold-induced shivering (up to 50% in one study), which requires vigilant monitoring during rapid cooling to avoid increased metabolic demand and potential complications (37).

Blinding considerations deserve mention. All included studies were inherently unblinded because participants undergoing exercise-induced hyperthermia cannot be blinded to active cooling interventions (e.g., ice-water-soaked tarpaulins) vs. passive rest. The high risk of performance bias identified by the RoB 2 tool reflects a field-wide methodological constraint rather than a flaw in individual study designs. This does not undermine objective outcomes such as core temperature measured by rectal probes or ingestible capsules, but it may affect subjective endpoints (e.g., thermal discomfort, perceived exertion). Readers should interpret secondary subjective outcomes with this limitation.

This study is not without limitations. First, the included trials were laboratory-based with small sample sizes (10–16 participants) and involved healthy young adults with exercise-induced hyperthermia (Tc typically < 40 °C and without central nervous system dysfuction), rather than patients with full clinical exertional heat stroke. Thus, our findings apply more directly to exercise-induced hyperthermia, and generalization to EHS requires caution. Second, all studies had high performance bias due to the inability to blind participants and personnel, potentially affecting subjective assessments. Third, secondary outcomes (including safety parameters and heart rate recovery) were incompletely reported across studies, limiting comparative analyses. Fourth, environmental settings varied considerably (controlled laboratory vs. outdoor field conditions with variable temperature, humidity, and airflow), contributing to statistical heterogeneity. Finally, despite comprehensive literature searches, the potential for publication bias due to unpublished negative results cannot be completely excluded.

Based on these findings and limitations, future research should focus on conducting high-quality, large-scale clinical trials to directly compare the efficacy and safety profiles of type A devices (with particular emphasis on waterproof tarpaulin methods) vs. passive cooling in authentic pre-hospital or simulated field settings. It is important to acknowledge that cold-water immersion remains the gold-standard cooling method for exertional heat stroke; therefore, future field studies should directly compare the cooling efficacy and practical feasibility of portable active cooling devices (particularly type A systems) against cold-water immersion. Additionally, further investigations are warranted to elucidate and optimize the mechanisms of action for type B devices. Finally, establishing a standardized core outcome set would facilitate consistent measurement and reporting across various clinical endpoints.

## 5 Conclusion

This meta-analysis reveals substantial heterogeneity in the effectiveness of portable active cooling devices for treating EHS or exercise-induced hyperthermia. Devices classified as Type A, which employ external refrigerants, demonstrate significantly enhanced cooling rates relative to passive cooling methods, particularly under elevated ambient temperatures, thereby warranting their consideration as effective interventions for field-based resuscitation. In contrast, current evidence fails to substantiate the superiority of wearable pre-cooled Type B devices over passive cooling when employed as standalone therapeutic modalities. These results offer crucial empirical support for refining pre-hospital EHS management protocols and developing evidence-based resource allocation frameworks, while simultaneously delineating key priorities for future device innovation and clinical investigation.

## Data Availability

The original contributions presented in the study are included in the article/Supplementary material, further inquiries can be directed to the corresponding author.
