# Supplementary material for: Comparative efficacy of portable active cooling vs. passive cooling for reducing core temperature in exertional heat stroke: a systematic review and meta-analysis
Source: Front Public Health. 2026 May 29;14:1811173. doi: 10.3389/fpubh.2026.1811173 (PMC13260578; doi:10.3389/fpubh.2026.1811173)
Supplement: Supplementary file 1 [file Table_1.docx]

**Table S1.** Information sources and search strategy.

| **Database** | **Search Strategy** |
| --- | --- |
| Embase | #15 #3 AND #7 AND #14  #14 #10 OR #13  #13 #11 OR #12  #12 'cross-over studies'/exp OR 'cross-over studies' OR ('cross over' AND ('studies'/exp OR studies)) OR 'cross over studies'/exp OR 'cross over studies' OR (cross AND over AND ('studies'/exp OR studies)) OR 'cross-over study'/exp OR 'cross-over study' OR ('cross over' AND ('study'/exp OR study)) OR 'studies, cross-over' OR (studies, AND 'cross over') OR 'study, cross-over' OR (('study,'/exp OR study,) AND 'cross over') OR 'crossover trials' OR (crossover AND trials) OR 'trial, crossover' OR (trial, AND crossover) OR 'trials, crossover' OR (trials, AND crossover) OR 'cross-over trials' OR ('cross over' AND trials) OR 'cross over trials' OR (cross AND over AND trials) OR 'trial, cross-over' OR (trial, AND 'cross over') OR 'trials, cross-over' OR (trials, AND 'cross over') OR 'crossover studies' OR (crossover AND ('studies'/exp OR studies)) OR 'crossover study'/exp OR 'crossover study' OR (crossover AND ('study'/exp OR study)) OR 'studies, crossover' OR (studies, AND crossover) OR 'study, crossover' OR (('study,'/exp OR study,) AND crossover) OR 'cross-over design'/exp OR 'cross-over design' OR ('cross over' AND ('design'/exp OR design)) OR 'cross over design'/exp OR 'cross over design' OR (cross AND over AND ('design'/exp OR design)) OR 'cross-over designs' OR ('cross over' AND designs) OR 'design, cross-over' OR (('design,'/exp OR design,) AND 'cross over') OR 'designs, cross-over' OR (designs, AND 'cross over') OR 'crossover design'/exp OR 'crossover design' OR (crossover AND ('design'/exp OR design)) OR 'crossover designs' OR (crossover AND designs) OR 'design, crossover' OR (('design,'/exp OR design,) AND crossover) OR 'designs, crossover' OR (designs, AND crossover)  #11 'crossover procedure'/exp  #10 #8 OR #9  #9 'controlled trial, randomized'/exp OR 'controlled trial, randomized' OR (controlled AND trial, AND randomized) OR 'randomised controlled study'/exp OR 'randomised controlled study' OR (randomised AND controlled AND ('study'/exp OR study)) OR 'randomised controlled trial'/exp OR 'randomised controlled trial' OR (randomised AND controlled AND ('trial'/exp OR trial)) OR 'randomized controlled study'/exp OR 'randomized controlled study' OR (randomized AND controlled AND ('study'/exp OR study)) OR 'trial, randomized controlled'/exp OR 'trial, randomized controlled' OR (trial, AND randomized AND controlled)  #8 'randomized controlled trial'/exp  #7 #4 OR #5 OR #6  #6 'cooling vest' OR (('cooling'/exp OR cooling) AND ('vest'/exp OR vest)) OR 'cooling garment' OR (('cooling'/exp OR cooling) AND garment) OR 'ice vest' OR (('ice'/exp OR ice) AND ('vest'/exp OR vest)) OR 'ice pack vest' OR (('ice'/exp OR ice) AND pack AND ('vest'/exp OR vest)) OR 'phase change material vest' OR (phase AND ('change'/exp OR change) AND material AND ('vest'/exp OR vest)) OR 'chemically activated cooling vest' OR (chemically AND activated AND ('cooling'/exp OR cooling) AND ('vest'/exp OR vest)) OR 'cold pack vest' OR (('cold'/exp OR cold) AND pack AND ('vest'/exp OR vest)) OR 'gel pack vest' OR (('gel'/exp OR gel) AND pack AND ('vest'/exp OR vest)) OR 'cooling jacket' OR (('cooling'/exp OR cooling) AND ('jacket'/exp OR jacket)) OR 'personal cooling system' OR (personal AND ('cooling'/exp OR cooling) AND system) OR 'wearable cooling' OR (wearable AND ('cooling'/exp OR cooling)) OR 'vest cooling' OR (('vest'/exp OR vest) AND ('cooling'/exp OR cooling))  #5 'tarp-assisted cooling' OR ('tarp assisted' AND ('cooling'/exp OR cooling)) OR 'tarp cooling' OR (tarp AND ('cooling'/exp OR cooling)) OR 'sheet cooling' OR (sheet AND ('cooling'/exp OR cooling)) OR 'water-assisted cooling' OR ('water assisted' AND ('cooling'/exp OR cooling)) OR 'ice sheet'/exp OR 'ice sheet' OR (('ice'/exp OR ice) AND sheet) OR 'wet sheet' OR (wet AND sheet) OR 'cooling blanket'/exp OR 'cooling blanket' OR (('cooling'/exp OR cooling) AND ('blanket'/exp OR blanket)) OR 'external cooling' OR (external AND ('cooling'/exp OR cooling)) OR 'conductive cooling' OR (conductive AND ('cooling'/exp OR cooling))  #4 'portable cooling' OR (portable AND ('cooling'/exp OR cooling)) OR 'field cooling' OR (field AND ('cooling'/exp OR cooling)) OR 'prehospital cooling' OR (prehospital AND ('cooling'/exp OR cooling)) OR 'evaporative cooling'/exp OR 'evaporative cooling' OR (evaporative AND ('cooling'/exp OR cooling)) OR 'conductive cooling' OR (conductive AND ('cooling'/exp OR cooling))  #3 #1 OR #2  #2 'heat strokes' OR (('heat'/exp OR heat) AND strokes) OR 'stroke, heat' OR (('stroke,'/exp OR stroke,) AND ('heat'/exp OR heat)) OR 'heatstroke'/exp OR heatstroke OR heatstrokes OR 'sun stroke'/exp OR 'sun stroke' OR (('sun'/exp OR sun) AND ('stroke'/exp OR stroke)) OR 'sunstroke'/exp OR sunstroke OR 'exertional heat stroke'/exp OR 'exertional heat stroke' OR (exertional AND ('heat'/exp OR heat) AND ('stroke'/exp OR stroke))  #1 'heat stroke'/exp |
| PubMed | ("Heat Stroke"[MeSH Terms] OR ("Heat Stroke"[MeSH Terms] OR ("heat"[All Fields] AND "stroke"[All Fields]) OR "Heat Stroke"[All Fields] OR ("heat"[All Fields] AND "strokes"[All Fields]) OR "heat strokes"[All Fields] OR ("Heat Stroke"[MeSH Terms] OR ("heat"[All Fields] AND "stroke"[All Fields]) OR "Heat Stroke"[All Fields] OR ("stroke"[All Fields] AND "heat"[All Fields]) OR "stroke heat"[All Fields]) OR ("Heat Stroke"[MeSH Terms] OR ("heat"[All Fields] AND "stroke"[All Fields]) OR "Heat Stroke"[All Fields] OR "heatstroke"[All Fields]) OR ("Heat Stroke"[MeSH Terms] OR ("heat"[All Fields] AND "stroke"[All Fields]) OR "Heat Stroke"[All Fields] OR "heatstrokes"[All Fields]) OR ("sunstroke"[MeSH Terms] OR "sunstroke"[All Fields] OR ("sun"[All Fields] AND "stroke"[All Fields]) OR "sun stroke"[All Fields]) OR ("sunstroke"[MeSH Terms] OR "sunstroke"[All Fields]) OR (("exertion"[All Fields] OR "exertional"[All Fields] OR "exertions"[All Fields]) AND ("Heat Stroke"[MeSH Terms] OR ("heat"[All Fields] AND "stroke"[All Fields]) OR "Heat Stroke"[All Fields])))) AND ((("portability"[All Fields] OR "portable"[All Fields] OR "portables"[All Fields]) AND ("cooled"[All Fields] OR "cooling"[All Fields] OR "coolings"[All Fields] OR "cools"[All Fields])) OR (("field"[All Fields] OR "field s"[All Fields] OR "fields"[All Fields]) AND ("cooled"[All Fields] OR "cooling"[All Fields] OR "coolings"[All Fields] OR "cools"[All Fields])) OR (("prehospital"[All Fields] OR "prehospitally"[All Fields]) AND ("cooled"[All Fields] OR "cooling"[All Fields] OR "coolings"[All Fields] OR "cools"[All Fields])) OR (("evaporable"[All Fields] OR "evaporate"[All Fields] OR "evaporated"[All Fields] OR "evaporates"[All Fields] OR "evaporating"[All Fields] OR "evaporation"[All Fields] OR "evaporations"[All Fields] OR "evaporative"[All Fields] OR "evaporatively"[All Fields] OR "evaporator"[All Fields] OR "evaporators"[All Fields]) AND ("cooled"[All Fields] OR "cooling"[All Fields] OR "coolings"[All Fields] OR "cools"[All Fields])) OR (("behavior"[MeSH Terms] OR "behavior"[All Fields] OR "conduct"[All Fields] OR "conducting"[All Fields] OR "conducts"[All Fields] OR "conductance"[All Fields] OR "conductances"[All Fields] OR "conducted"[All Fields] OR "conductibility"[All Fields] OR "conduction"[All Fields] OR "conductions"[All Fields] OR "conductive"[All Fields] OR "conductively"[All Fields] OR "conductivities"[All Fields] OR "conductivity"[All Fields]) AND ("cooled"[All Fields] OR "cooling"[All Fields] OR "coolings"[All Fields] OR "cools"[All Fields])) OR (("Tarp-assisted"[All Fields] AND ("cooled"[All Fields] OR "cooling"[All Fields] OR "coolings"[All Fields] OR "cools"[All Fields])) OR (("tarp"[Supplementary Concept] OR "tarp"[All Fields]) AND ("cooled"[All Fields] OR "cooling"[All Fields] OR "coolings"[All Fields] OR "cools"[All Fields])) OR (("sheet"[All Fields] OR "sheet s"[All Fields] OR "sheeted"[All Fields] OR "sheeting"[All Fields] OR "sheetings"[All Fields] OR "sheets"[All Fields]) AND ("cooled"[All Fields] OR "cooling"[All Fields] OR "coolings"[All Fields] OR "cools"[All Fields])) OR ("Water-assisted"[All Fields] AND ("cooled"[All Fields] OR "cooling"[All Fields] OR "coolings"[All Fields] OR "cools"[All Fields])) OR ("ice cover"[MeSH Terms] OR ("ice"[All Fields] AND "cover"[All Fields]) OR "ice cover"[All Fields] OR ("ice"[All Fields] AND "sheet"[All Fields]) OR "ice sheet"[All Fields]) OR ("Wet"[All Fields] AND ("sheet"[All Fields] OR "sheet s"[All Fields] OR "sheeted"[All Fields] OR "sheeting"[All Fields] OR "sheetings"[All Fields] OR "sheets"[All Fields])) OR (("cooled"[All Fields] OR "cooling"[All Fields] OR "coolings"[All Fields] OR "cools"[All Fields]) AND ("blanket"[All Fields] OR "blanketing"[All Fields] OR "blankets"[All Fields])) OR (("external"[All Fields] OR "externally"[All Fields] OR "externals"[All Fields]) AND ("cooled"[All Fields] OR "cooling"[All Fields] OR "coolings"[All Fields] OR "cools"[All Fields])) OR (("behavior"[MeSH Terms] OR "behavior"[All Fields] OR "conduct"[All Fields] OR "conducting"[All Fields] OR "conducts"[All Fields] OR "conductance"[All Fields] OR "conductances"[All Fields] OR "conducted"[All Fields] OR "conductibility"[All Fields] OR "conduction"[All Fields] OR "conductions"[All Fields] OR "conductive"[All Fields] OR "conductively"[All Fields] OR "conductivities"[All Fields] OR "conductivity"[All Fields]) AND ("cooled"[All Fields] OR "cooling"[All Fields] OR "coolings"[All Fields] OR "cools"[All Fields]))) OR ((("cooled"[All Fields] OR "cooling"[All Fields] OR "coolings"[All Fields] OR "cools"[All Fields]) AND "Vest"[All Fields]) OR (("cooled"[All Fields] OR "cooling"[All Fields] OR "coolings"[All Fields] OR "cools"[All Fields]) AND ("clothing"[MeSH Terms] OR "clothing"[All Fields] OR "garment"[All Fields] OR "garments"[All Fields] OR "garment s"[All Fields])) OR (("ice"[Supplementary Concept] OR "ice"[All Fields] OR "ice"[MeSH Terms]) AND "Vest"[All Fields]) OR (("ice"[Supplementary Concept] OR "ice"[All Fields] OR "ice"[MeSH Terms]) AND "pack"[All Fields] AND "Vest"[All Fields]) OR (("phase"[All Fields] OR "phase s"[All Fields] OR "phases"[All Fields]) AND ("change"[All Fields] OR "changed"[All Fields] OR "changes"[All Fields] OR "changing"[All Fields] OR "changings"[All Fields]) AND ("material"[All Fields] OR "material s"[All Fields] OR "materials"[All Fields]) AND "Vest"[All Fields]) OR (("chemical"[All Fields] OR "chemical s"[All Fields] OR "chemically"[All Fields] OR "chemicals"[All Fields]) AND ("activable"[All Fields] OR "activate"[All Fields] OR "activated"[All Fields] OR "activates"[All Fields] OR "activating"[All Fields] OR "activation"[All Fields] OR "activations"[All Fields] OR "activator"[All Fields] OR "activator s"[All Fields] OR "activators"[All Fields] OR "active"[All Fields] OR "actived"[All Fields] OR "actively"[All Fields] OR "actives"[All Fields] OR "activities"[All Fields] OR "activity s"[All Fields] OR "activitys"[All Fields] OR "exercise"[MeSH Terms] OR "exercise"[All Fields] OR "activity"[All Fields]) AND ("cooled"[All Fields] OR "cooling"[All Fields] OR "coolings"[All Fields] OR "cools"[All Fields]) AND "Vest"[All Fields]) OR (("common cold"[MeSH Terms] OR ("common"[All Fields] AND "cold"[All Fields]) OR "common cold"[All Fields] OR "cold"[All Fields] OR "cold temperature"[MeSH Terms] OR ("cold"[All Fields] AND "temperature"[All Fields]) OR "cold temperature"[All Fields]) AND "pack"[All Fields] AND "Vest"[All Fields]) OR ("Gel"[All Fields] AND "pack"[All Fields] AND "Vest"[All Fields]) OR (("cooled"[All Fields] OR "cooling"[All Fields] OR "coolings"[All Fields] OR "cools"[All Fields]) AND ("jacket"[All Fields] OR "jacketed"[All Fields] OR "jacketing"[All Fields] OR "jackets"[All Fields])) OR (("person s"[All Fields] OR "personable"[All Fields] OR "personableness"[All Fields] OR "personal"[All Fields] OR "personalisation"[All Fields] OR "personalise"[All Fields] OR "personalised"[All Fields] OR "personalising"[All Fields] OR "personality"[MeSH Terms] OR "personality"[All Fields] OR "personalities"[All Fields] OR "personality s"[All Fields] OR "personalization"[All Fields] OR "personalize"[All Fields] OR "personalized"[All Fields] OR "personalizes"[All Fields] OR "personalizing"[All Fields] OR "personally"[All Fields] OR "personals"[All Fields] OR "persons"[MeSH Terms] OR "persons"[All Fields] OR "person"[All Fields]) AND ("cooled"[All Fields] OR "cooling"[All Fields] OR "coolings"[All Fields] OR "cools"[All Fields]) AND ("system"[All Fields] OR "system s"[All Fields] OR "systems"[All Fields])) OR (("wearability"[All Fields] OR "wearable"[All Fields] OR "wearables"[All Fields]) AND ("cooled"[All Fields] OR "cooling"[All Fields] OR "coolings"[All Fields] OR "cools"[All Fields])) OR ("Vest"[All Fields] AND ("cooled"[All Fields] OR "cooling"[All Fields] OR "coolings"[All Fields] OR "cools"[All Fields])))) AND ("Randomized Controlled Trial"[Publication Type] OR ("randomized controlled trials as topic"[MeSH Terms] OR ("randomized"[All Fields] AND "controlled"[All Fields] AND "trials"[All Fields] AND "topic"[All Fields]) OR "randomized controlled trials as topic"[All Fields] OR ("controlled"[All Fields] AND "trial"[All Fields] AND "randomized"[All Fields]) OR "controlled trial randomized"[All Fields] OR (("random allocation"[MeSH Terms] OR ("random"[All Fields] AND "allocation"[All Fields]) OR "random allocation"[All Fields] OR "randomization"[All Fields] OR "randomized"[All Fields] OR "random"[All Fields] OR "randomisation"[All Fields] OR "randomisations"[All Fields] OR "randomise"[All Fields] OR "randomised"[All Fields] OR "randomising"[All Fields] OR "randomizations"[All Fields] OR "randomize"[All Fields] OR "randomizes"[All Fields] OR "randomizing"[All Fields] OR "randomness"[All Fields] OR "randoms"[All Fields]) AND "controlled"[All Fields] AND ("studies"[All Fields] OR "study"[All Fields] OR "study s"[All Fields] OR "studying"[All Fields] OR "studys"[All Fields])) OR ("Randomized Controlled Trial"[Publication Type] OR "randomized controlled trials as topic"[MeSH Terms] OR "randomised controlled trial"[All Fields] OR "Randomized Controlled Trial"[All Fields]) OR (("random allocation"[MeSH Terms] OR ("random"[All Fields] AND "allocation"[All Fields]) OR "random allocation"[All Fields] OR "randomization"[All Fields] OR "randomized"[All Fields] OR "random"[All Fields] OR "randomisation"[All Fields] OR "randomisations"[All Fields] OR "randomise"[All Fields] OR "randomised"[All Fields] OR "randomising"[All Fields] OR "randomizations"[All Fields] OR "randomize"[All Fields] OR "randomizes"[All Fields] OR "randomizing"[All Fields] OR "randomness"[All Fields] OR "randoms"[All Fields]) AND "controlled"[All Fields] AND ("studies"[All Fields] OR "study"[All Fields] OR "study s"[All Fields] OR "studying"[All Fields] OR "studys"[All Fields])) OR (("clinical trials as topic"[MeSH Terms] OR ("clinical"[All Fields] AND "trials"[All Fields] AND "topic"[All Fields]) OR "clinical trials as topic"[All Fields] OR "trial"[All Fields] OR "trial s"[All Fields] OR "trialed"[All Fields] OR "trialing"[All Fields] OR "trials"[All Fields]) AND ("random allocation"[MeSH Terms] OR ("random"[All Fields] AND "allocation"[All Fields]) OR "random allocation"[All Fields] OR "randomization"[All Fields] OR "randomized"[All Fields] OR "random"[All Fields] OR "randomisation"[All Fields] OR "randomisations"[All Fields] OR "randomise"[All Fields] OR "randomised"[All Fields] OR "randomising"[All Fields] OR "randomizations"[All Fields] OR "randomize"[All Fields] OR "randomizes"[All Fields] OR "randomizing"[All Fields] OR "randomness"[All Fields] OR "randoms"[All Fields]) AND "controlled"[All Fields])) OR ("Cross-Over Studies"[MeSH Terms] OR ("Cross-Over Studies"[MeSH Terms] OR ("cross over"[All Fields] AND "studies"[All Fields]) OR "Cross-Over Studies"[All Fields] OR ("cross"[All Fields] AND "over"[All Fields] AND "studies"[All Fields]) OR "Cross-Over Studies"[All Fields] OR ("Cross-Over Studies"[MeSH Terms] OR ("cross over"[All Fields] AND "studies"[All Fields]) OR "Cross-Over Studies"[All Fields] OR ("cross"[All Fields] AND "over"[All Fields] AND "study"[All Fields]) OR "cross over study"[All Fields]) OR ("Cross-Over Studies"[MeSH Terms] OR ("cross over"[All Fields] AND "studies"[All Fields]) OR "Cross-Over Studies"[All Fields] OR ("studies"[All Fields] AND "cross"[All Fields] AND "over"[All Fields]) OR "studies cross over"[All Fields]) OR ("Cross-Over Studies"[MeSH Terms] OR ("cross over"[All Fields] AND "studies"[All Fields]) OR "Cross-Over Studies"[All Fields] OR ("study"[All Fields] AND "cross"[All Fields] AND "over"[All Fields]) OR "study cross over"[All Fields]) OR ("Cross-Over Studies"[MeSH Terms] OR ("cross over"[All Fields] AND "studies"[All Fields]) OR "Cross-Over Studies"[All Fields] OR ("crossover"[All Fields] AND "trials"[All Fields]) OR "crossover trials"[All Fields]) OR ("Cross-Over Studies"[MeSH Terms] OR ("cross over"[All Fields] AND "studies"[All Fields]) OR "Cross-Over Studies"[All Fields] OR ("trial"[All Fields] AND "crossover"[All Fields]) OR "trial crossover"[All Fields]) OR ("Cross-Over Studies"[MeSH Terms] OR ("cross over"[All Fields] AND "studies"[All Fields]) OR "Cross-Over Studies"[All Fields] OR ("trials"[All Fields] AND "crossover"[All Fields]) OR "trials crossover"[All Fields]) OR ("Cross-Over Studies"[MeSH Terms] OR ("cross over"[All Fields] AND "studies"[All Fields]) OR "Cross-Over Studies"[All Fields] OR ("cross"[All Fields] AND "over"[All Fields] AND "trials"[All Fields]) OR "cross over trials"[All Fields]) OR ("Cross-Over Studies"[MeSH Terms] OR ("cross over"[All Fields] AND "studies"[All Fields]) OR "Cross-Over Studies"[All Fields] OR ("cross"[All Fields] AND "over"[All Fields] AND "trials"[All Fields]) OR "cross over trials"[All Fields]) OR ("Cross-Over Studies"[MeSH Terms] OR ("cross over"[All Fields] AND "studies"[All Fields]) OR "Cross-Over Studies"[All Fields] OR ("trial"[All Fields] AND "cross"[All Fields] AND "over"[All Fields]) OR "trial cross over"[All Fields]) OR ("Cross-Over Studies"[MeSH Terms] OR ("cross over"[All Fields] AND "studies"[All Fields]) OR "Cross-Over Studies"[All Fields] OR ("trials"[All Fields] AND "cross"[All Fields] AND "over"[All Fields]) OR "trials cross over"[All Fields]) OR ("Cross-Over Studies"[MeSH Terms] OR ("cross over"[All Fields] AND "studies"[All Fields]) OR "Cross-Over Studies"[All Fields] OR ("crossover"[All Fields] AND "studies"[All Fields]) OR "crossover studies"[All Fields]) OR ("Cross-Over Studies"[MeSH Terms] OR ("cross over"[All Fields] AND "studies"[All Fields]) OR "Cross-Over Studies"[All Fields] OR ("crossover"[All Fields] AND "study"[All Fields]) OR "crossover study"[All Fields]) OR ("Cross-Over Studies"[MeSH Terms] OR ("cross over"[All Fields] AND "studies"[All Fields]) OR "Cross-Over Studies"[All Fields] OR ("studies"[All Fields] AND "crossover"[All Fields]) OR "studies crossover"[All Fields]) OR ("Cross-Over Studies"[MeSH Terms] OR ("cross over"[All Fields] AND "studies"[All Fields]) OR "Cross-Over Studies"[All Fields] OR ("study"[All Fields] AND "crossover"[All Fields]) OR "study crossover"[All Fields]) OR ("Cross-Over Studies"[MeSH Terms] OR ("cross over"[All Fields] AND "studies"[All Fields]) OR "Cross-Over Studies"[All Fields] OR ("cross"[All Fields] AND "over"[All Fields] AND "design"[All Fields]) OR "cross over design"[All Fields]) OR ("Cross-Over Studies"[MeSH Terms] OR ("cross over"[All Fields] AND "studies"[All Fields]) OR "Cross-Over Studies"[All Fields] OR ("cross"[All Fields] AND "over"[All Fields] AND "design"[All Fields]) OR "cross over design"[All Fields]) OR ("Cross-Over Studies"[MeSH Terms] OR ("cross over"[All Fields] AND "studies"[All Fields]) OR "Cross-Over Studies"[All Fields] OR ("cross"[All Fields] AND "over"[All Fields] AND "designs"[All Fields]) OR "cross over designs"[All Fields]) OR ("Cross-Over Studies"[MeSH Terms] OR ("cross over"[All Fields] AND "studies"[All Fields]) OR "Cross-Over Studies"[All Fields] OR ("design"[All Fields] AND "cross"[All Fields] AND "over"[All Fields]) OR "design cross over"[All Fields]) OR ("Cross-Over Studies"[MeSH Terms] OR ("cross over"[All Fields] AND "studies"[All Fields]) OR "Cross-Over Studies"[All Fields] OR ("designs"[All Fields] AND "cross"[All Fields] AND "over"[All Fields])) OR ("Cross-Over Studies"[MeSH Terms] OR ("cross over"[All Fields] AND "studies"[All Fields]) OR "Cross-Over Studies"[All Fields] OR ("crossover"[All Fields] AND "design"[All Fields]) OR "crossover design"[All Fields]) OR ("Cross-Over Studies"[MeSH Terms] OR ("cross over"[All Fields] AND "studies"[All Fields]) OR "Cross-Over Studies"[All Fields] OR ("crossover"[All Fields] AND "designs"[All Fields]) OR "crossover designs"[All Fields]) OR ("Cross-Over Studies"[MeSH Terms] OR ("cross over"[All Fields] AND "studies"[All Fields]) OR "Cross-Over Studies"[All Fields] OR ("design"[All Fields] AND "crossover"[All Fields]) OR "design crossover"[All Fields]) OR ("Cross-Over Studies"[MeSH Terms] OR ("cross over"[All Fields] AND "studies"[All Fields]) OR "Cross-Over Studies"[All Fields] OR ("designs"[All Fields] AND "crossover"[All Fields]) OR "designs crossover"[All Fields])))) |
| Web of science | #9 #1 AND #5 AND #8  #8 #6 OR #7  #7 TS=(Cross-Over Studies) OR TS=(Cross Over Studies) OR TS=(Cross-Over Study) OR TS=(Studies, Cross-Over) OR TS=(Study, Cross-Over) OR TS=(Crossover Trials) OR TS=(Trial, Crossover) OR TS=(Trials, Crossover) OR TS=(Cross-Over Trials) OR TS=(Cross Over Trials) OR TS=(Trial, Cross-Over) OR TS=(Trials, Cross-Over) OR TS=(Crossover Studies) OR TS=(Crossover Study) OR TS=(Studies, Crossover) OR TS=(Study, Crossover) OR TS=(Cross-Over Design) OR TS=(Cross Over Design) OR TS=(Cross-Over Designs) OR TS=(Design, Cross-Over) OR TS=(Designs, Cross-Over) OR TS=(Crossover Design) OR TS=(Crossover Designs) OR TS=(Design, Crossover) OR TS=(Designs, Crossover)  #6 TS=(Randomized Controlled Trial ) OR TS=(controlled trial, randomized) OR TS=(randomised controlled study) OR TS=(randomised controlled trial) OR TS=(randomized controlled study) OR TS=(trial, randomized controlled) OR TS=(randomized controlled trial)  #5 #2 OR #3 OR #4  #4 TS=(Cooling vest) OR TS=(Cooling garment) OR TS=(Ice vest) OR TS=(Ice pack vest) OR TS=(Phase change material vest) OR TS=(Chemically activated cooling vest) OR TS=(Cold pack vest) OR TS=(Gel pack vest) OR TS=(Cooling jacket) OR TS=(Personal cooling system) OR TS=(Wearable cooling) OR TS=(Vest cooling)  #3 TS=(Tarp-assisted cooling) OR TS=(Tarp cooling) OR TS=(Sheet cooling) OR TS=(Water-assisted cooling) OR TS=(Ice sheet) OR TS=(Wet sheet) OR TS=(Cooling blanket) OR TS=(External cooling) OR TS=(Conductive cooling)  #2 TS=(Portable cooling) OR TS=(Field cooling) OR TS=(Prehospital cooling) OR TS=(Evaporative cooling) OR TS=(Conductive cooling)  #1 TS=(Heat stroke) OR TS=(Heat Strokes) OR TS=(Stroke, Heat) OR TS=(Heatstroke) OR TS=(Heatstrokes) OR TS=(sun stroke) OR TS=(Sunstroke) OR TS=(Exertional heat stroke) |
| Cochrance | #1 MeSH descriptor: [Heat Stroke] explode all trees  #2 (Heat Strokes):ti,ab,kw OR (Stroke, Heat):ti,ab,kw OR (Heatstroke):ti,ab,kw OR (Heatstrokes):ti,ab,kw OR (sun stroke):ti,ab,kw  #3 (Sunstroke):ti,ab,kw OR (Exertional heat stroke):ti,ab,kw  #4 #1 OR #2 OR #3  #5 (Portable cooling):ti,ab,kw OR (Field cooling):ti,ab,kw OR (Prehospital cooling):ti,ab,kw OR (Evaporative cooling):ti,ab,kw OR (Conductive cooling):ti,ab,kw  #6 (Tarp-assisted cooling):ti,ab,kw OR (Tarp cooling):ti,ab,kw OR (Sheet cooling):ti,ab,kw OR (Water-assisted cooling):ti,ab,kw OR (Ice sheet):ti,ab,kw  #7 (Wet sheet):ti,ab,kw OR (Cooling blanket):ti,ab,kw OR (External cooling):ti,ab,kw OR (Conductive cooling):ti,ab,kw  #8 (Cooling vest):ti,ab,kw OR (Cooling garment):ti,ab,kw OR (Ice vest):ti,ab,kw OR (Ice pack vest):ti,ab,kw OR (Phase change material vest):ti,ab,kw  #9 (Chemically activated cooling vest):ti,ab,kw OR (Cold pack vest):ti,ab,kw OR (Gel pack vest):ti,ab,kw OR (Cooling jacket):ti,ab,kw OR (Personal cooling system):ti,ab,kw  #10 (Wearable cooling):ti,ab,kw OR (Vest cooling):ti,ab,kw  #11 #5 OR #6 OR #7 OR #8 OR #9 OR #10  #12 MeSH descriptor: [Randomized Controlled Trial] explode all trees  #13 (controlled trial, randomized):ti,ab,kw OR (randomised controlled study):ti,ab,kw OR (randomised controlled trial):ti,ab,kw OR (randomized controlled study):ti,ab,kw OR (trial, randomized controlled):ti,ab,kw  #14 #12 OR #13  #15 MeSH descriptor: [Cross-Over Studies] explode all trees  #16 (Cross Over Studies):ti,ab,kw OR (Cross-Over Study):ti,ab,kw OR (Studies, Cross-Over):ti,ab,kw OR (Study, Cross-Over):ti,ab,kw OR (Crossover Trials):ti,ab,kw  #17 (Trial, Crossover):ti,ab,kw OR (Trials, Crossover):ti,ab,kw OR (Cross-Over Trials):ti,ab,kw OR (Cross Over Trials):ti,ab,kw OR (Trial, Cross-Over):ti,ab,kw  #18 (Trials, Cross-Over):ti,ab,kw OR (Crossover Studies):ti,ab,kw OR (Crossover Study):ti,ab,kw OR (Studies, Crossover):ti,ab,kw OR (Study, Crossover):ti,ab,kw  #19 (Cross-Over Design):ti,ab,kw OR (Cross Over Design):ti,ab,kw OR (Cross-Over Designs):ti,ab,kw OR (Design, Cross-Over):ti,ab,kw OR (Designs, Cross-Over):ti,ab,kw  #20 (Crossover Design):ti,ab,kw OR (Crossover Designs):ti,ab,kw OR (Design, Crossover):ti,ab,kw OR (Designs, Crossover):ti,ab,kw  #21 #15 OR #16 OR #17 OR #18 OR #19 OR #20  #22 #14 OR #21  #23 #4 AND #11 AND #22 |
| Medline | #9 #1 AND #5 AND #8  #8 #6 OR #7  #7 TS=(Cross-Over Studies) OR TS=(Cross Over Studies) OR TS=(Cross-Over Study) OR TS=(Studies, Cross-Over) OR TS=(Study, Cross-Over) OR TS=(Crossover Trials) OR TS=(Trial, Crossover) OR TS=(Trials, Crossover) OR TS=(Cross-Over Trials) OR TS=(Cross Over Trials) OR TS=(Trial, Cross-Over) OR TS=(Trials, Cross-Over) OR TS=(Crossover Studies) OR TS=(Crossover Study) OR TS=(Studies, Crossover) OR TS=(Study, Crossover) OR TS=(Cross-Over Design) OR TS=(Cross Over Design) OR TS=(Cross-Over Designs) OR TS=(Design, Cross-Over) OR TS=(Designs, Cross-Over) OR TS=(Crossover Design) OR TS=(Crossover Designs) OR TS=(Design, Crossover) OR TS=(Designs, Crossover)  #6 TS=(Randomized Controlled Trial ) OR TS=(controlled trial, randomized) OR TS=(randomised controlled study) OR TS=(randomised controlled trial) OR TS=(randomized controlled study) OR TS=(trial, randomized controlled) OR TS=(randomized controlled trial)  #5 #2 OR #3 OR #4  #4 TS=(Cooling vest) OR TS=(Cooling garment) OR TS=(Ice vest) OR TS=(Ice pack vest) OR TS=(Phase change material vest) OR TS=(Chemically activated cooling vest) OR TS=(Cold pack vest) OR TS=(Gel pack vest) OR TS=(Cooling jacket) OR TS=(Personal cooling system) OR TS=(Wearable cooling) OR TS=(Vest cooling)  #3 TS=(Tarp-assisted cooling) OR TS=(Tarp cooling) OR TS=(Sheet cooling) OR TS=(Water-assisted cooling) OR TS=(Ice sheet) OR TS=(Wet sheet) OR TS=(Cooling blanket) OR TS=(External cooling) OR TS=(Conductive cooling)  #2 TS=(Portable cooling) OR TS=(Field cooling) OR TS=(Prehospital cooling) OR TS=(Evaporative cooling) OR TS=(Conductive cooling)  #1 TS=(Heat stroke) OR TS=(Heat Strokes) OR TS=(Stroke, Heat) OR TS=(Heatstroke) OR TS=(Heatstrokes) OR TS=(sun stroke) OR TS=(Sunstroke) OR TS=(Exertional heat stroke) |
